# Functional Integration of mRNA Translational Control Programs

**DOI:** 10.3390/biom5031580

**Published:** 2015-07-21

**Authors:** Melanie C. MacNicol, Chad E. Cragle, Karthik Arumugam, Bruno Fosso, Graziano Pesole, Angus M. MacNicol

**Affiliations:** 1 Department of Neurobiology and Developmental Sciences, University of Arkansas for Medical Sciences, Little Rock, AR 72205, USA; E-Mail: Mel@UAMS.edu; 2 Center for Translational Neuroscience, University of Arkansas for Medical Sciences, Little Rock, AR 72205, USA; 3 Interdisciplinary BioSciences Graduate Program, University of Arkansas for Medical Sciences, Little Rock, AR 72205, USA; E-Mail: CCragle@uams.edu; 4 Department of Physiology and Biophysics, University of Arkansas for Medical Sciences, Little Rock, AR 72205, USA; E-Mail: Karthik.Arumugam@crg.eu; 5 Institute of Biomembranes and Bioenergetics, National Research Council, Bari 70126, Italy; E-Mail: bruno.fosso@gmail.com; 6 Department of Biosciences, Biotechnology and Biopharmaceutics, University of Bari, Bari 70125, Italy; 7 Winthrop P. Rockefeller Cancer Institute, University of Arkansas for Medical Sciences, Little Rock, AR 72205, USA

**Keywords:** Musashi, CPEB, RNA-binding protein, mRNA translation, combinatorial control, mRNP, regulation

## Abstract

Regulated mRNA translation plays a key role in control of cell cycle progression in a variety of physiological and pathological processes, including in the self-renewal and survival of stem cells and cancer stem cells. While targeting mRNA translation presents an attractive strategy for control of aberrant cell cycle progression, mRNA translation is an underdeveloped therapeutic target. Regulated mRNAs are typically controlled through interaction with multiple RNA binding proteins (RBPs) but the mechanisms by which the functions of distinct RBPs bound to a common target mRNA are coordinated are poorly understood. The challenge now is to gain insight into these mechanisms of coordination and to identify the molecular mediators that integrate multiple, often conflicting, inputs. A first step includes the identification of altered mRNA ribonucleoprotein complex components that assemble on mRNAs bound by multiple, distinct RBPs compared to those recruited by individual RBPs. This review builds upon our knowledge of combinatorial control of mRNA translation during the maturation of oocytes from *Xenopus laevis*, to address molecular strategies that may mediate RBP diplomacy and conflict resolution for coordinated control of mRNA translational output. Continued study of regulated ribonucleoprotein complex dynamics promises valuable new insights into mRNA translational control and may suggest novel therapeutic strategies for the treatment of disease.

## 1. Regulated mRNA Translation and Control of Cell Cycle

Control of mRNA translation is critical for regulation of cell cycle progression during key physiological and pathological processes [1,2,3,4,5,6,7,8]. Translation is regulated at both the global level, through control of components that are required for translation of the majority of cellular mRNAs, and at the level of specific, targeted subsets of mRNAs. Targeted control of specific mRNAs is mediated through RNA binding proteins (RBPs) or through miRNA-directed complexes that target regulatory elements typically located within the mRNA 5' or 3' untranslated regions (UTR). Targeted control of mRNA translation is particularly important during the growth, self-renewal, and survival of stem cells [9,10,11,12] and contributes pathologically to the self-renewal and survival of cancer stem cells, a subpopulation of cancer cells that, through unique growth and survival properties, evade conventional treatment regimens to contribute to tumor recurrence and metastasis [13,14,15,16,17,18,19,20,21,22,23,24,25,26]. While mRNA translation could present an attractive strategy for control of physiological and pathological stem cell self-renewal, mRNA translation is underdeveloped, therapeutically, due to a dearth of identified “druggable” targets [27].

Recent technical advances have provided unprecedented insights into the ribonucleoprotein complex components that are involved in mRNA translation [28,29,30,31,32]. However, an understanding of mechanisms that specify the unique translational output of individual mRNA species is less well developed. Over 800 RBPs have now been identified in the human genome, and utilization of various cross-linking and mass spectrometry strategies have provided “interactome” maps of RBP occupancy on cellular mRNAs [33,34]. The interactome studies provide a snapshot of RBP occupancy on cellular mRNAs, but do not necessarily provide information on the dynamics of RBP interactions or RBP-associated cofactor assemblies over time, or in response to environmental cues. The challenge for the field in the years ahead is to gain insight into how RBPs coordinate their activities, particularly in regard to the modifications of their behavior that occur on complex mRNA substrates that harbor binding sites for other distinct RBPs with different regulatory control and/or antagonistic function(s).

**Figure 1:**
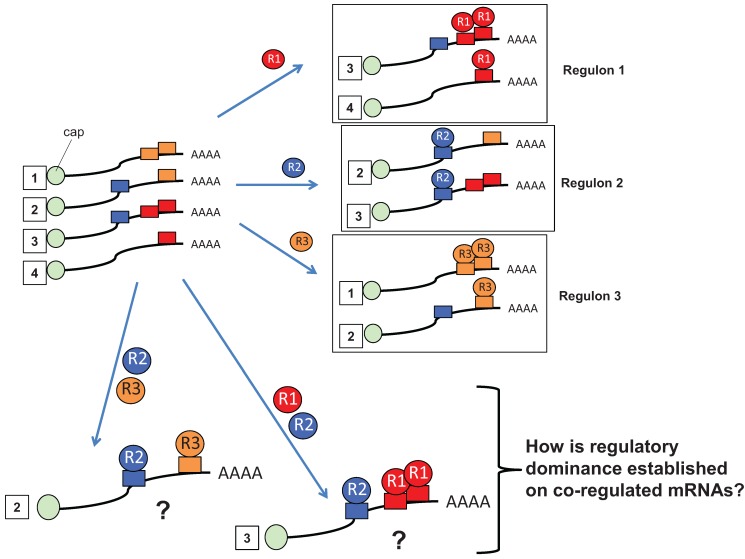
Coordinated control of mRNA subsets. The four cytoplasmic mRNAs shown (labeled with numbered white squares) form three RNA regulons that are determined by the binding of RBPs (labeled R1, R2 and R3) to specific sequence elements within the mRNAs. These interactions lead to co-regulation of distinct mRNAs within each RNA regulon (adapted from [35]). The fate of co-regulated, co-occupied mRNAs is less clear when mRNAs possess binding sites for multiple RBPs with antagonistic action (e.g., bound by both R1 and R2 concurrently (mRNA 3) or R2 and R3 (mRNA 2).

It has been recognized that coordination of distinct mRNA species encoding functionally related protein products is mediated by shared RBPs and their associated co-factors in higher order mRNA ribonucleoprotein (mRNP) complexes (Figure 1). These coordinated mRNA subsets have been termed “regulons” [1,35]. The regulon model posits that mRNAs are partitioned into distinct regulons by virtue of interaction with a specific RBP. An aspect of this model that has not been fully addressed concerns the situation where a cell expresses multiple interacting RBPs with antagonistic function. In this case, it is not clear, mechanistically, how one RBP can establish functional dominance on co-regulated target mRNAs in lieu of competitive binding for the same target site(s). Resolution of conflicting regulatory inputs may result in dominance of one RBP, partitioning of an mRNA into multiple regulons or an altered fate and partitioning to a new regulon (see mRNAs 2 and 3, Figure 1). Resolving conflicting co-regulatory inputs in *cis* is an emergent issue as there is growing experimental evidence that mRNAs with common RBP interactions are differentially regulated within the same cell context [36,37,38,39,40]. In this review, we seek to extend the RNA regulon model by addressing possible molecular mechanisms by which distinct RBPs can influence, in a context-dependent manner, the translational activity of a shared target mRNA. We will discuss the mechanisms of combinatorial control of mRNA translation during the maturation of oocytes from *Xenopus laevis,* to provide perspective into molecular strategies mediating diplomacy and conflict resolution on co-regulated mRNA transcripts pertinent to mammalian mRNA translational control.

In the *Xenopus* model system, immature oocytes are arrested in a dormant state until activated to re-enter the cell cycle in response to the steroid hormone, progesterone. Synthesis of the proteins required for cell cycle re-entry and progression is achieved through selective activation and translation of pre-existing mRNAs [41]. Translation of targeted mRNAs proceeds in a sequential, temporally controlled fashion that is coordinated through *cis* elements within the mRNA and the *trans-*acting RBP interactome that is specifically recruited to these elements [42]. Specifically, the mRNA encoding Ringo, an atypical cyclin-dependent kinase activator, is translated through relief of repression exerted by the Pumilio RBP [43,44], followed by translation of the mRNA encoding the MAP kinase activator, Mos, through action of the Musashi RBP [36,38], culminating in the translation of the mRNA encoding the cyclin-dependent kinase activator Cyclin B1 through the CPEB1- and CPEB4 RBPs [45,46]. The Zygotic Arrest (Zar1 and Zar2) RBPs have also been shown to act early within this program to mediate translational control, although the requirement for Zar function for oocyte cell cycle progression has not yet been established [47,48]. Each step in this process is dependent upon correct completion of the previous step, thus ensuring coordinated control of cell cycle progression [49,50].

**Figure 2:**
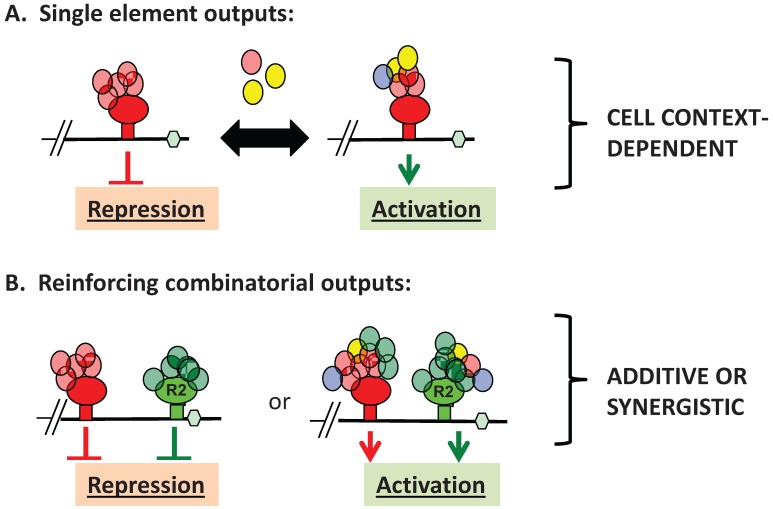

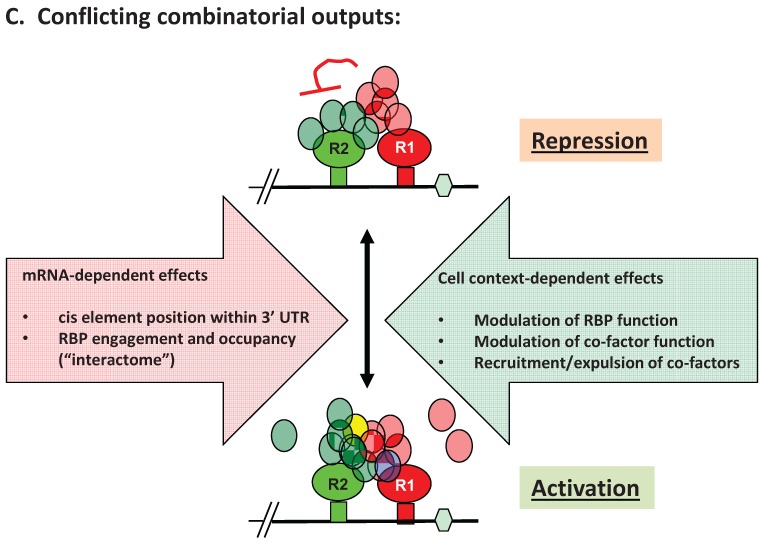
mRNA translational diplomacy—integration of multiple regulatory inputs. (**A**) When an mRNA is regulated by a single RBP (R1, large red oval) binding to its target site (red square), co-associated factors (small red ovals) mediate control of translation through interaction with 5' initiation factors, elongation factors, ribosomal constituents or 3' end processing factors as part of a higher order mRNP complex. In response to intrinsic or extrinsic cellular cues (Black arrow), remodeling of the mRNP occurs (e.g., recruitment of additional factors (yellow oval), expulsion of existing components (red oval), modification of existing component (blue oval)), resulting in altered control of translation. Hexagon, polyadenylation signal; (**B**) Control of mRNA translation often involves multiple distinct RBPs (R1 and R2, large red and red oval, respectively) interacting with the same target mRNA. When the RBPs exert similar function in any given cellular context, their activities lead to additive or synergistic regulation of translational output; (**C**) When the bound RBPs exert opposing functions, mRNA translational output reflects integration of conflicting RBP inputs. A combinatorial assembly of effector co-factors would permit recruitment, expulsion and/or modification of the higher order mRNP complex in response to changing cellular cues, resulting in mRNA-specific translational output. In this example the activity of a repressor RBP (R1, large red oval) and associated co-factors (small red ovals) overrides the activator function of a neighboring RBP (R2, large green oval) and its associated co-factors (small green ovals). However, modulation of R1 and/or R2 directly, modulation of co-factor function and/or association attenuates the ability of R1 to inhibit R2 activator activity. Interaction of the distinct RBPs (R1 and R2) thus results in mRNA- and cell context-dependent combinatorial assembly of recruited co-factors specific to the RBP occupancy.

Coordination of translational control during oocyte maturation is mediated in part through the dual functionality of participating RBPs. The Musashi and CPEB1 RBPs have been shown to exert distinct RNA control functions, either repression or activation of translation, in a cell context-specific fashion (Figure 2A) [51,52]. Distinct signaling pathways have been identified in *Xenopus* that mediate the cell context-dependent phosphorylation events that control the functional switch of individual RBPs. Musashi phosphorylation is initiated through Ringo-dependent, cyclin-dependent kinase (CDK) signaling leading to the translation of the mRNA encoding the Mos proto-oncogene [36,38,53]. De-repression and activation of CPEB1-mediated translation is initiated through Mos-dependent MAP kinase signaling, leading to the translation of the cyclin B1 and CPEB4 mRNAs [45,54,55]. Through the subsequent downstream action of cyclin B-CDK signaling and the peptidyl-prolyl *cis-trans* isomerase, Pin1, CPEB1 undergoes partial degradation [56,57,58]. Maintenance of CPEB-mediated translation activation after initiation of CPEB1 degradation is mediated by the compensatory action of CPEB4 [46]. As will be discussed below, many mRNAs contain sites for two or more of these regulatory RBPs, so the dependency of progression through each step in this process upon an activator generated in the previous step, provides a mechanism of temporal enforcement and coordination to control cell cycle progression.

## 2. Multiple RBP Input Is a Prevalent Regulatory Mechanism for Control of mRNA Translation

Examination of the limited subset of regulated *Xenopus* mRNAs encoding key cell cycle control proteins hitherto characterized, reveals that their 3' UTRs contain multiple, distinct *cis* element RBP binding sites (reviewed in [42]), including the Pumilio binding element (PBE); the Musashi binding element (MBE); the Zar-targeted Translational Control Sequence (TCS); and the CPEB1 and CPEB4 cytoplasmic polyadenylation element (CPE). Two immediate issues arise concerning mRNA co-regulatory inputs: (1) how common is it for an mRNA 3' UTR to contain sites for multiple, distinct RBPs? and (2) How does the cellular translation machinery decipher combinatorial regulatory inputs?

To better determine the concurrence of multiple elements on a global scale, we assessed their representation in 26,511 non-redundant *Xenopus* mRNA 3' UTRs collected in the 3' UTR database [59]. This analysis found that approximately 2/3rds (17,190 out of 26,511) of all *Xenopus* 3' UTRs contain multiple, distinct, regulatory motifs (Table 1, discounting 3' UTRs containing only a single element: PBE only (273), MBE only (2771), CPE only (2274), TCS only (282); or no element matches (3721)). Notably, MBE and CPE co-representation was particularly common, occurring in 57% (15,101 out of 26,511) of all mRNA 3' UTRs. A similar analysis of human and murine mRNAs revealed MBE and CPE co-representation in 57% and 54% of all human and murine 3' UTRs, respectively (Table 1). Although no positional dependence has been reported for MBE functionality, CPE mediated translational activation is restricted with regard to distance from the polyadenylation signal [60] and so the absolute number of co-regulated mRNAs may be somewhat lower than predicted. Nonetheless, we predict that a significant proportion of vertebrate mRNAs are subject to co-regulatory inputs from both Musashi and CPEB families of RBPs. Furthermore, the overall the proportion of co-regulated mRNAs is expected to increase significantly when one considers target sites for the over 800 identified RBPs, as well as miRNA target sites.

**Table 1:** Vertebrate mRNA 3' UTRs commonly contain multiple distinct regulatory elements.

HOMO SAPIENS (66,974 Total 3' UTRs)		MUS MUSCULUS (26,420 Total 3' UTRs)		XENOPUS (26,511 Total 3' UTRs)	
Sequence Motif Combinations	Matching 3' UTRs	Sequence Motif Combinations	Matching 3' UTRs	Sequence Motif Combinations	Matching 3' UTRs
PBE	15,156	PBE	5444	PBE	6312
& MBE	14,437	& MBE	5136	& MBE	5521
NOT MBE	719	NOT MBE	308	NOT MBE	791
& CPE	13,536	& CPE	4693	& CPE	5427
NOT CPE	1620	NOT CPE	751	NOT CPE	885
& TCS	9462	& TCS	3101	& TCS	2738
NOT TCS	5694	NOT TCS	2343	NOT TCS	3574
NOT (MBE OR CPE)	409	NOT (MBE OR CPE)	174	NOT (MBE OR CPE)	314
NOT (MBE OR TCS)	613	NOT (MBE OR TCS)	272	NOT (MBE OR TCS)	638
NOT (CPE OR TCS)	1287	NOT (CPE OR TCS)	606	NOT (CPE OR TCS)	735
NOT (MBE OR CPE OR TCS)	373	NOT (MBE OR CPE OR TCS)	162	NOT (MBE OR CPE OR TCS)	273
MBE	50,101	MBE	20,079	MBE	18,912
& PBE	14,437	& PBE	5136	& PBE	5521
NOT PBE	35,664	NOT PBE	14,943	NOT PBE	13,391
& CPE	37,483	& CPE	14,215	& CPE	15,101
NOT CPE	12,618	NOT CPE	5864	NOT CPE	3811
& TCS	21,858	& TCS	8358	& TCS	6861
NOT TCS	28,243	NOT TCS	11,721	NOT TCS	12,051
NOT (PBE OR CPE)	11,407	NOT (PBE OR CPE)	5287	NOT (PBE OR CPE)	3240
NOT (PBE OR TCS)	23,162	NOT (PBE OR TCS)	9650	NOT (PBE OR TCS)	9115
NOT (CPE OR TCS)	10,670	NOT (CPE OR TCS)	4790	NOT (CPE OR TCS)	3233
NOT (PBE OR CPE OR TCS)	9756	NOT (PBE OR CPE OR TCS)	4346	NOT (PBE OR CPE OR TCS)	2771
CPE	41,567	CPE	15,698	CPE	18,383
& PBE	13,536	& PBE	4693	& PBE	5427
NOT PBE	28,031	NOT PBE	11,005	NOT PBE	12,956
& MBE	37,483	& MBE	14,215	& MBE	15,101
NOT MBE	4084	NOT MBE	1483	NOT MBE	3282
& TCS	20,638	& TCS	7518	& TCS	6926
NOT TCS	20,929	NOT TCS	8180	NOT TCS	11,457
NOT (PBE OR MBE)	3774	NOT (PBE OR MBE)	1349	NOT (PBE OR MBE)	2805
NOT (PBE OR TCS)	16,522	NOT (PBE OR TCS)	6443	NOT (PBE OR TCS)	8618
NOT (MBE OR TCS)	3356	NOT (MBE OR TCS)	1249	NOT (MBE OR TCS)	2639
NOT (PBE OR MBE OR TCS)	3116	NOT (PBE OR MBE OR TCS)	1139	NOT (PBE OR MBE OR TCS)	2274
TCS	23,235	TCS	8880	TCS	7827
& PBE	9462	& PBE	3101	& PBE	2738
NOT PBE	13,773	NOT PBE	5779	NOT PBE	5089
& MBE	21,858	& MBE	8358	& MBE	6861
NOT MBE	1377	NOT MBE	522	NOT MBE	966
& CPE	20,638	& CPE	7518	& CPE	6926
NOT CPE	2597	NOT CPE	1362	NOT CPE	901
NOT (PBE OR MBE)	1271	NOT (PBE OR MBE)	486	NOT (PBE OR MBE)	813
NOT (PBE OR CPE)	2264	NOT (PBE OR CPE)	1217	NOT (PBE OR CPE)	751
NOT (MBE OR CPE)	649	NOT (MBE OR CPE)	288	NOT (MBE OR CPE)	323
NOT (PBE OR MBE OR CPE)	613	NOT (PBE OR MBE OR CPE)	276	NOT (PBE OR MBE OR CPE)	282
NO MATCH	11,767	NO MATCH	4408	NO MATCH	3721

The 3' UTR database [59] was queried by using PatSearch [61] for the representation of regulatory motifs, alone or in combination using the following parameters: Pumilio binding element (PBE) of sequence: UGUANAUA; Musashi binding element (MBE) of sequence: RU_1-3_AGU or GUAG; Translational Control Element (TCS) of sequence: WUURUCU; Cytoplasmic polyadenylation element (CPE) of sequence: UUUU(A_1–3_)U, UUUUAAGU, UUUUACU, or UUUUCAU. The number of 3' UTRs containing the indicated motif, or combination of motifs is indicated. For these analyses, 66,974 human, 26,420 murine and 26,511 *Xenopus* non-redundant 3' UTR sequences were assessed. The results do not indicate how many times the element occurs on the same 3' UTR. This information is available in Supplementary Table 1, along with element position within each 3' UTR. When the search motifs were randomly shuffled and assessed against the *Xenopus* database entries, the absolute numbers of CPE and PBE matches greatly exceed their shuffled control sequence (44,341 CPE matches *vs.* 29,404 for the shuffled motif, 8790 PBE matches *vs.* 3551 for the shuffled motif). The MBE motif was underrepresented compared to the randomly shuffled motif (49,762 MBE matches *vs.* 74,053 for the shuffled motif), perhaps reflecting selection against the presence of the potential TAG STOP codon sequence within the motif. Given their frequency variance from their randomly shuffled motif, the PBE, MBE, and CPE motifs identify candidate 3’ UTRs with high confidence. The TCS motif by contrast did not vary significantly from a randomly shuffled variant (11,239 TCS matches *vs.* 11,734 for the shuffled counterpart) and so caution should be exercised when assessing identified TCS-containing 3' UTRs.

## 3. Advantages of mRNA Combinatorial Control

Unlike transcriptional control mechanisms, the regulated template (mRNA) pre-exists in the cell, often at high copy number, specifically to enable a rapid and robust cellular responses to extrinsic and intrinsic cues. In this “primed” system, a benefit of co-regulation would be reinforcement of regulatory control. In the immature oocyte, TCS, CPE, and PBEs can all direct translational repression of a target mRNA. An mRNA with multiple distinct elements would be less likely to experience translational “leak” and would be subject to a greater level of repression than that generated by a single type of element, while positive reinforcement of translational activation by multiple elements may augment the translational output of the mRNA (Figure 2B) [40,62,63,64,65].

In addition to cooperative functional integration, co-regulation also allows regulatory conflicts to impact translational output (Figure 2C). Thus, two distinct mRNAs that share a common RBP may be subject to *differential* mRNA translational control within the same cell type. A number of examples of such differential control have been recently reported. These include the early *vs.* late translational activation of the MBE and CPE containing *Xenopus* Mos and cyclin B1 mRNAs in *Xenopus* oocytes [36,37,38], CPEB1-dependent repression of the cyclin B1 mRNA and coincident CPEB1-dependent translational activation of the Dnmt1 mRNA in HeLa cells [40], as well as the Musashi-dependent repression of TGFβR1 mRNA and coincident activation of the SMAD3 mRNA in K562 cells [39].

The physiological advantages of co-regulation by distinct RBPs or miRNAs include mRNA-specific fine-tuning of translational output of target mRNAs in both temporal and spatial patterns in response to a variety of cellular stimuli. Control of an RNA through multiple RBPs that are independently regulated by distinct signaling pathways [42] could support translation over a longer, more sustained period than could be achieved through control of any one RBP alone. Conversely, control through a combination of an activating plus an inhibitory RBP could result in a more truncated, acute burst of translation than may be achievable through a single regulatory mechanism. The Mos mRNA is targeted by both Musashi and CPEB1, and is repressed in immature oocytes through CPEB1-mediated repression. In response to progesterone, the Mos mRNA undergoes early translation, mediated through Musashi activation, and sustained late translation, mediated through CPEB activation [36,38]. Thus, co-regulation can be used to enforce stage-specific regulation of mRNA translational control, either through contemporaneous action leading to acute elevated or truncated output, or through sequential action to generate longer, sustained responses.

Co-regulation could also allow selective translational control coupled with targeted sub-cellular localization. An example of such a regulatory principle in mammalian cells is the regulation of CAT-1 mRNA translation by miRNA and HuR control. In response to cellular stress, HuR is relocated from the nucleus to the cytoplasm where it can displace miR-122 targeting the CAT-1 mRNA in repressive P-bodies, promoting release of CAT-1 mRNA from P-bodies and translation [66]. In this case, the modulation of translation involves HuR oligomerization on the CAT-1 mRNA and displacement of miR-122 repressive RISC complexes [67]. In addition, HuR and AUF1 are often considered to exert opposite translational control of shared AU-rich containing target mRNAs through competitive binding. A more complex mechanism is suggested, however, by the observation of concurrent HuR and AUF-1 binding to non-overlapping sites in the nuclear compartment, while HuR and AUF-1 tend to bind to target mRNAs individually in the cytoplasm [68]. The redistribution of regulatory binding was suggested to be a consequence of several factors including relative RBP abundance, subcellular localization and cellular stress signaling.

A consideration when assessing the likely output of combinatorial control concerns the relative positional effect of each element within the same mRNA. As a case in point, the position of MBE and CPEs, relative to each other and relative to common functional elements such as the polyadenylation hexanucleotide (Hex) has been observed to greatly influence translation. In *Xenopus*, the function of factors recruited by Musashi at the MBE have been shown to act in a dominant manner over factors recruited by CPEB1, resulting in early translational activation as long as the CPE does not overlap the Hex. However, when a CPE does overlap the Hex, the CPEB inhibitory complex dominates over neighboring Musashi-recruited stimulatory complexes (reviewed in [42]). Similarly, the cryptic function of dual TCS elements to direct early translation of the Wee1 mRNA is suppressed by flanking CPE sequences in the Wee1 mRNA [63,69].

Under these conditions of contemporaneous functional conflict, some means of diplomacy must exist to integrate and coordinate opposing regulatory inputs. A possible mechanism could involve the physical association of effector complexes recruited by the individual RBPs via “bridging” or adaptor proteins and the selective silencing or expulsion of key conflicted factors, resulting in mRNA-specific assemblies of higher order mRNP complexes. Such regulatory assemblies could be dynamically remodeled in response to altered cellular signaling. The translational status of an mRNA would thus reflect the integrated sum of the effects of all retained co-factors recruited by the distinct cohort of RBPs that are linked to the target the mRNA in a cell-context-dependent manner (Figure 2C).

## 4. RBP-Specific Co-Factor Interactions

Before we can address components that may bridge between distinct RBPs, we should first consider what factors are known to bind to the individual RBPs. In immature *Xenopus* oocytes, multiple distinct complex assemblies have been identified as contributing to CPEB1-mediated repression (reviewed in [70]). CPEB1 has been proposed to exert repression of bound mRNAs via association with the eIF4G-binding protein Maskin, which functions as a competitive inhibitor of the 5' cap-binding initiation factor eIF4E for eIF4G interaction thus preventing translational initiation [71]. CPEB1 has also been shown to interact with the eIF4E cap binding variant, eIF4Eb and the eIF4E binding protein, eIF4E-T [72]. The interaction of eIF4Eb with eIF4E-T attenuates interaction with eIF4G thus repressing the mRNA through sequestration of the 5' cap. In addition, CPEB1 has been shown to interact with both the atypical poly[A] polymerase GLD2 and the functionally opposing poly[A] ribonuclease, PARN. In immature oocytes, the activity of PARN counteracts that of GLD2 thus maintaining a short poly[A] tail and translational repression of CPE-containing mRNAs [73]. Each of these repression complexes are altered in response to progesterone stimulation to allow CPEB1 to functionally switch to direct activation of target mRNA translation. CPEB activation has been shown to involve phosphorylation of Maskin and of 4E-T to disrupt these repressor complexes [72,74]. Similarly, PARN is expelled from phosphorylated CPEB1 thereby disrupting the CPEB1/GLD2/PARN complex to allow unfettered action of GLD2, extension of the poly[A] tail of target mRNAs and activation of translation [73].

The co-factor requirements for Musashi function are less well understood. Like CPEB1, Musashi1 has been shown to interact with both PARN and GLD2, with the GLD2 interaction being necessary for the translational activation of target mRNAs during oocyte maturation [75]. Unlike CPEB1 however, Musashi1 co-association with PARN is not disrupted in progesterone-stimulated oocytes, suggesting distinct sensitivities to inhibition by PARN. In mammalian cells, Musashi-exerted repression has been reported to be mediated through interaction with a member of the poly[A] binding protein family, PABPC. It has been suggested that interaction occurs within a C-terminal domain of Musashi1 and that this interaction interferes with eIF4G function and large ribosome subunit recruitment [76]. Interestingly, PABPC has been previously implicated as an activator of mRNA translation [77], and so further work is required to delineate the unique aspects of the mammalian PABPC interaction that facilitate Musashi repressor activity. Like CPEB1, Musashi can switch from a repressor to an activator of target mRNA translation in response to cell stimulation [52,78], and it is possible that PABPC plays a cell context-dependent role in mediating this switch in Musashi function. Alternatively, regulation of Musashi function by PABPC and by GLD2 may be mutually exclusive, as these two proteins both bind the same C-terminal region of the Musashi1 protein [75]. Such mutually exclusive interactions support a model in which selective binding to PABPC or to GLD2 would result in repression or activation of target mRNA translation, respectively. Such partitioning of co-associated factors could result in Musashi target mRNA populations associated with either repressor or activator complexes within the same cell.

Several recent studies have described the situation in which RBPs mediate different functions on distinct RNAs [39,40]. The mechanism by which these distinct functions are exerted by the same RBP is unknown. Possible mechanisms to stabilize distinct assemblies would be through the use of a retention barrier such as differential subcellular partitioning or through interaction with distinct isoforms of RBPs that bind common mRNA target elements. In this latter regard, the Musashi2 isoform diverges from Musashi1 in the PABPC/GLD2 interaction domain. Unlike Musashi1, mammalian Musashi2 does not interact with GLD2 and may also not interact with PABPC [75,79]. Further distinctions in protein associations between these Musashi isoforms have been reported including association of the miRNA biogenesis regulatory factor, Lin28 exclusively with Musashi1 [80] and the Sox2 transcriptional regulator of stem cell pluripotency with only Musashi2 [81], suggesting that Musashi1- and Musashi2-associated mRNP complexes may be comprised of non-redundant proteins that can mediate distinct regulatory and/or functional actions of these isoforms upon target RNAs [82]. However, in the case of hematopoietic cells, Musashi2 is the primary isoform expressed [83,84], and so the observed differential control of TGFβR1 and SMAD3 mRNA translation [39] cannot be explained by differential binding of Musashi1 or Musashi2 isoforms. A possible alternative mechanism would be through altered Musashi complex composition or stability as a consequence of mRNA-specific recruitment of co-factors, as dictated by additional distinct RBP regulatory elements within the same mRNA. Thus, the differential control of distinct target mRNAs is likely mediated through proximity to neighboring RBPs and the influence of their associated co-factors. The influence of neighboring RBP complexes would be expected to be subject to positional effects, potentially explaining the observed role of relative position in mediating MBE or CPE dominance in mRNAs containing both regulatory elements, as discussed earlier. Moreover, an implicit assumption is that the mRNA-specific regulatory components would be subject to dynamic cell context-dependent remodeling as mediated through modification of the RBP, modification of associated co-factors and/or through the recruitment or expulsion of co-factors (Figure 2C).

## 5. Experimental Evidence for Adaptive Regulatory Assemblies in the Control of Translation

If distinct RBP regulatory assemblies physically interact it should be possible to identify common, potentially shared, co-factors that facilitate communication and integration of functionally distinct RBPs. Using Musashi and CPEB1 as exemplars, reports of several shared components exist in the literature. Both GLD2 and PARN are found in Musashi and CPEB1 complexes in immature oocytes. CPEB1 has also been reported to interact with embryonic poly[A] binding protein [85], the most abundant member of the PABP family expressed in immature oocytes [86]. Although it is not yet known if Musashi also associates with ePAB, common interaction with the PABP family may provide another link between CPEB1 and Musashi proteins.

**Figure 3:**
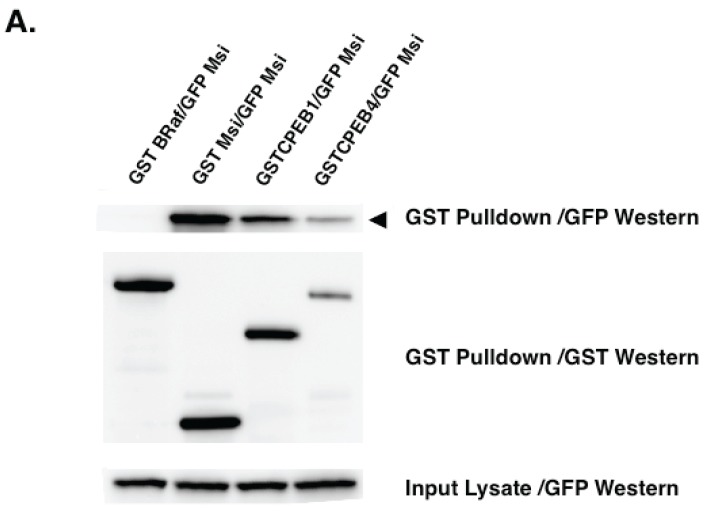

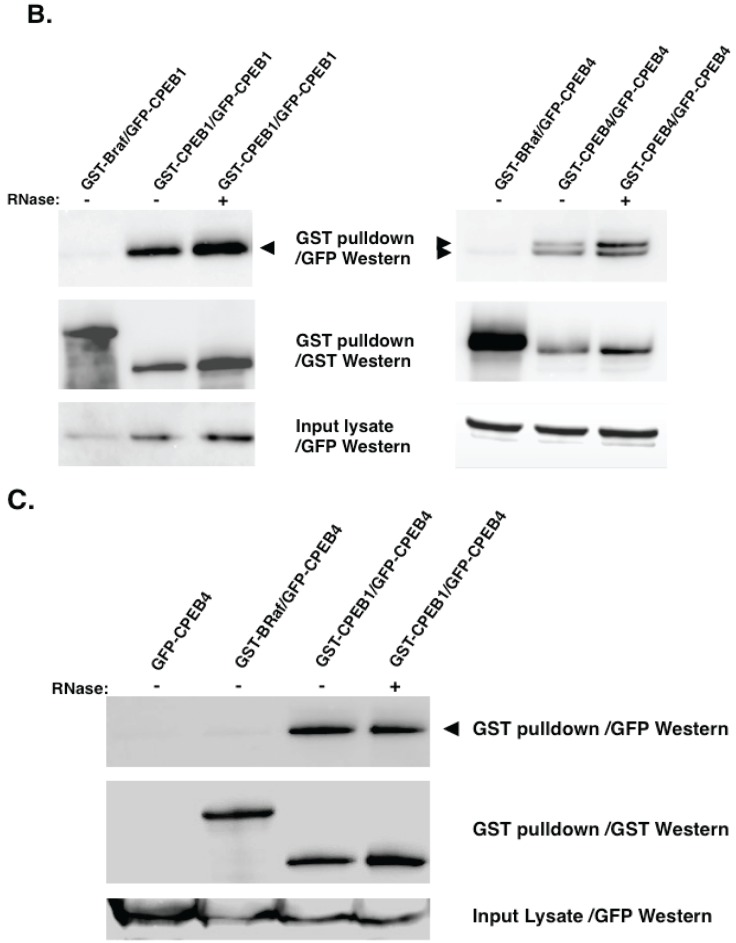
Musashi co-association with other RBPs is RNA-independent (**A**) Forty oocytes were co-injected with mRNA encoding GFP-Musashi1 (Msi) and either GST-BRaf, GST-Msi, GST-CPEB1 or GST-CPEB4. The GFP tagged mammalian Musashi1 protein construct [75], and GST-tagged human CPEB1 [91] have been described previously. The CPEB4 protein construct was generated by a PCR mediated subclone of KIAA 1673 (Origene Technologies, Inc., Rockville, MD USA) into the pXen2 vector [92]. *In vitro* transcribed RNA was prepared for each construct and injected into immature oocytes. The injected oocytes were incubated overnight to express the introduced proteins then lysed. Lysates were then subjected to GST-pulldown and treatment with RNase1 as described [75]. Associations were visualized by western blotting. GST-XMsi1, GST-CPEB1 and GST-CPEB4 associate with GFP-Msi in an RNase1 independent manner, while the GST-BRaf does not (upper panel, arrowhead); (**B**) Oocytes were injected with the indicated mRNA combinations and CPEB1 (left panel) or CPEB4 (right panel) oligomerization assessed by GFP western blotting (arrowhead, upper panels) in the presence or absence of added Rnase1, essentially as described in panel (**a**) above. No co-association was seen with BRaf. The expressed CPEB4 protein runs as a doublet in these experiments; (**C**) Oocytes were injected with the indicated mRNA combinations and CPEB1:CPEB4 co-association assessed by GFP western blotting, as described in panels (**A**,**B**).

To assess possible Musashi and CPEB interactions more directly, we performed co-association analyses. Musashi1 was found to associate with both CPEB1 and CPEB4 (Figure 3A). These assays were performed in the presence of RNase 1, and so the persistence of these associations precludes the co-precipitation of these RBPs through simple co-occupancy of a common mRNA and instead indicate protein:protein interactions between Musashi and CPEB1 as well as Musashi1 and CPEB4. In this same analysis, Musashi1 was shown to form Musashi1:Musashi1 homoligomers (Figure 3A). No association of Musashi1 was observed with GST tagged BRaf (an activator of MAP kinase signaling, [87]), demonstrating specificity of the Musashi1:Musashi1 and Musashi1:CPEB interactions. Our analyses also revealed CPEB1:CPEB1 and CPEB4:CPEB4 homodimer formation (Figure 3B, left and right panels, respectively), consistent with prior findings [88,89]. Interestingly, we also observed CPEB1:CPEB4 heterodimers (Figure 3C), further adding to the range and complexity of mRNP formation between distinct RBPs.

To determine if the Musashi interaction with CPEB1 or with CPEB4 was a consequence of direct binding, we employed the yeast two hybrid protein:protein association assay. This assay indicated that while Musashi1:Musashi1 homodimerization was the result of direct association, Musashi1 did not associate directly with either CPEB1 or CPEB4 (Table 2). We conclude that Musashi1 interaction with CPEB1 and CPEB4 is mediated indirectly through intermediary bridging protein(s). Further studies will determine the extent to which such bridging proteins coordinate the functions of Musashi1, CPEB1 and CPEB4.

The coordinated dynamic assembly and disassembly of shared protein complexes would allow communication between RBPs for reinforcement of similar functions and resolution of opposing functions. Such communication may be mediated through prevention of co-factor association within a shared mRNP in one cell context and facilitation of association in response to extracellular cues. There is evidence for such communication between Musashi1 and CPEB1. Musashi-directed mRNA translational activation is reduced in the absence of activating phosphorylation of CPEB1, which would preclude dissociation of the CPEB1:PARN complex [37]. An interpretation of this finding is that although disruption of the Musashi1:PARN association does not appear to be required for Musashi function [75], failure to disrupt the CPEB1 repressor complex impedes Musashi function on mRNAs occupied by both RBPs. One could envision that an inhibitory factor may be stabilized by the CPEB1 repressor complex and through proximity with the Musashi1 activation complex, act to attenuate Musashi function. Perturbation of one RBP may thus have unexpected pleiotropic consequences on cellular mRNA translation due to functional integration within higher order mRNP complexes that bridge multiple RBPs. Further, the regulon model may be viewed similarly to the competing endogenous mRNA hypothesis, where perturbation of expression levels of one mRNA can modulate expression of other unrelated mRNAs due to sharing of miRNA regulatory sequences [90], or in this case, shared RBP effector complexes. Such pleiotropic perturbations complicate interpretation of the cause and effect in the cellular changes that occur in response to overexpression or knockdown of RBPs in experimental or pathological conditions.

**Table 2:** Musashi1 interaction with CPEB1 and CPEB4 is indirect.

pGBK DNA Binding Fusion Vector	pACT Transcriptional Activation Fusion Vector	Interaction?
Empty	Musashi1	No
Musashi1	Empty	No
Musashi1	CPEB1	No
Musashi1	CPEB4	No
Musashi1	Musashi1	Yes

The yeast two-hybrid assay (Matchmaker II; Clontech) was used to identify Musashi-interacting proteins, as per the manufacturer’s instructions. Mammalian Musashi1 [75], CPEB1 [91] or CPEB4 (KIAA 1673, Origene), were subcloned by PCR into the indicated vector and transformed into yeast strain AH109. Colony growth was selected on -Leu-Trp-His plates in the presence of 1mM 3-AT (3-amino-1,2,4-triazole). Any colony growth after 5 days was scored as positive for the tested protein:protein interaction.

## 6. Conclusions

Regulated mRNA translation is required to control the transitions between phases of the vertebrate cell cycle, including cell cycle entry and cell cycle exit. An understanding of the complex coordination of the components that make up mRNA-specific regulatory complexes presents both a challenge and an opportunity in control of mRNA translation. The challenge is in identifying, among the many distinct proteins that converge upon mRNA translation, those that may be amenable to manipulation (e.g., “druggable” targets). The opportunity lies with the fact that if the key regulator of translation of a specific mRNA is not a good target for manipulation, knowledge of the mRNP complex that interacts with the regulator may enable a work-around by manipulation of co-associated proteins. A better understanding of the mechanisms by which mRNA translation is modulated and coordinated may lead to development of novel approaches for control of cell cycle progression and cell fate, including anti-cancer therapy and stem cell lineage manipulation for regenerative medicine.

## Supplementary Files

Supplementary File 1
